# Caffeinated Gel Ingestion Enhances Jump Performance, Muscle Strength, and Power in Trained Men

**DOI:** 10.3390/nu11040937

**Published:** 2019-04-25

**Authors:** Sandro Venier, Jozo Grgic, Pavle Mikulic

**Affiliations:** 1 Faculty of Kinesiology, University of Zagreb, Zagreb 10000, Croatia; veniersandro@gmail.com; 2 Institute for Health and Sport (IHES), Victoria University, Melbourne 3011, Australia; jozo.grgic@vu.live.edu.au

**Keywords:** caffeine, ergogenic aid, resistance training, isokinetic testing

## Abstract

We aimed to explore the effects of caffeinated gel ingestion on neuromuscular performance in resistance-trained men. The participants (*n* = 17; mean ± standard deviation (SD): age 23 ± 2 years, height 183 ± 5 cm, body mass 83 ± 11 kg) completed two testing conditions that involved ingesting a caffeinated gel (300 mg of caffeine) or placebo. The testing outcomes included: (1) vertical jump height in the squat jump (SJ) and countermovement jump (CMJ); (2) knee extension and flexion peak torque and average power at angular velocities of 60°·s^−1^ and 180°·s^−1^; (3) barbell velocity in the bench press with loads corresponding to 50%, 75%, and 90% of one-repetition maximum (1RM); and (4) peak power output in a test on a rowing ergometer. Compared to the placebo, caffeine improved: (1) SJ (*p* = 0.039; Cohen’s *d* effect size (*d*) = 0.18; +2.9%) and CMJ height (*p* = 0.011; *d* = 0.18; +3.3%); (2) peak torque and average power in the knee extensors at both angular velocities (*d* ranged from 0.21 to 0.37; percent change from +3.5% to +6.9%), peak torque (*p* = 0.034; *d* = 0.24; +4.6%), and average power (*p* = 0.015; *d* = 0.32; +6.7%) at 60°·s^−1^ in the knee flexors; (3) barbell velocity at 50% 1RM (*p* = 0.021; *d* = 0.33; +3.5%), 75% 1RM (*p* < 0.001; *d* = 0.42; +5.4%), and 90% 1RM (*p* < 0.001; *d* = 0.59, +12.0%). We conclude that the ingestion of caffeinated gels may acutely improve vertical jump performance, strength, and power in resistance-trained men.

## 1. Introduction

In the general population, caffeine is a widely consumed food constituent [1]. Caffeine consumption is also widespread among athletes, likely due to its performance-enhancing effects on exercise [2]. In most of the studies that examine the effects of caffeine ingestion on exercise performance, the participants ingest caffeine administered in the form of a capsule and wait 60 min before starting the exercise session [3,4]. This waiting period is used with the idea that plasma levels of caffeine reach their peak values ~60 min following the ingestion of a caffeine-containing capsule [5]. 

In recent years, however, several studies have explored the effects of alternate sources of caffeine on exercise performance [3]. Some of the alternate sources of caffeine include chewing gums, bars, gels, mouth rinses, energy drinks, aerosols, and coffee [3,6,7]. These sources attracted the attention of researchers, given that they may provide rapid absorption of caffeine in the body. For example, following the consumption of a caffeine-containing gum, increases in caffeine levels in plasma occur within 5 min [8]. This rapid absorption may lead to a faster ergogenic effect, which subsequently may be useful in many situations in sport and in exercise settings.

Wickham and Spriet [3] highlighted that only two studies thus far have examined the effects of caffeinated gels on exercise performance; one reported an ergogenic effect of caffeine on 2000-m rowing-ergometer performance [9], while another stated that caffeine ingestion did not enhance intermittent sprint performance [10]. Due to the scarce and conflicting studies examining the effects of caffeinated gels on exercise performance, it is evident that further research with this source of caffeine is warranted.

Two recent meta-analyses reported that caffeine ingestion acutely enhances muscle strength, as assessed by isokinetic peak torque and jumping performance [11,12]. In both meta-analyses, all included studies explored the effects of caffeine administered in the form of a capsule or liquid. 

In resistance exercise, caffeine ingestion may acutely increase muscle strength, muscle endurance, and muscle power [13]. However, the effects of caffeine on muscle power in resistance exercise have been explored the least. Grgic et al. [13] highlighted only four studies [14,15,16,17] that have explored the effect of caffeine on power (as assessed by barbell velocity). Grgic et al. [13] suggest that caffeine may have a considerable performance-enhancing effect on barbell velocity in resistance exercise; however, the authors also noted the need for future research on the topic. Given that all four studies that examined the effects of caffeine on muscle power in resistance exercise used caffeine in the form of a capsule, it remains unclear if comparable effects may be observed with caffeinated gel as a source of caffeine. While studies are exploring the effects of caffeine on resistance exercise administered in alternate forms such as coffee and chewing gums [6,7,18], there is a lack of studies utilizing caffeinated gels.

An additional limitation of the current body of evidence that explored the effects of caffeine on power is that almost all studies used performance tests that involved a specific body region in isolation (e.g., upper-body in the bench press exercise). Currently, there is a need for studies that measure power output during exercise tests that require simultaneous coordinated activity of the upper- and lower-body musculature. 

This study aimed to explore the effects of caffeinated gel ingestion on: (1) jump performance; (2) isokinetic strength and power of the knee extensor and knee flexor muscles; (3) upper-body power; and (4) whole-body power, in a sample of resistance-trained men. We hypothesized that ingesting a caffeinated gel would acutely enhance exercise performance in all of the employed performance tests compared to the placebo.

## 2. Materials and Methods 

### 2.1. Study Design

This study employed a randomized, crossover, double-blind, counterbalanced study design. In the first exercise session, participants were familiarized with the performance tests. Following this familiarization session, the participants were randomized to two experimental conditions: caffeinated gel and placebo gel. The dose of caffeinated gel (Smart 1 Energizer Gel, Science in Sport) contained 88 g of carbohydrates and 300 mg of caffeine. The placebo gel (Go Isotonic Energy Gel, Science in Sport) contained the same amount of carbohydrates without any caffeine. Therefore, the only difference in the provided gels was the amount of caffeine. 

After ingesting either the placebo or caffeinated gel, the participants were given 10 min to warm-up before the testing session started. All testing sessions were conducted in the morning hours (between 7:00 and 9:00 a.m.) for all participants. The day before each testing session, the participants were requested to maintain their general nutritional and sleep habits, and not to perform any vigorous physical activity. Additionally, the participants were asked to refrain from any caffeine ingestion after 6:00 p.m. on the days before the two experimental conditions. To facilitate this process of caffeine restriction, the participants were provided with a comprehensive list of the most common food and drink products containing caffeine. The participants were also instructed not to ingest any food or drinks (other than plain water) upon waking up; that is, they came to the laboratory in a fasted state. Adherence to these guidelines was established before the start of each testing session. The testing sessions were separated by no less than three and no more than six days. The reliability of the outcomes analyzed in the exercise protocol was established on a pilot sample of five participants that repeated the exercise protocol on two occasions, three days apart (Table 1). 

### 2.2. Participants

The following inclusion criteria was set for this study: (1) apparently healthy men, aged 18–45 years, without any current muscular injuries or other physical limitations; and (2) resistance-trained, defined as having at least one year of resistance exercise experience with a minimal weekly training frequency of two times per week, and by having the ability to successfully lift at least 100% of their current body mass in the bench press exercise. 

A power analysis performed prior to the study initiation using the G*Power software indicated that the required sample size for this study is 12 participants. The parameters employed in this analysis were as follows: expected effect *f* of 0.20 (for barbell velocity in the bench press exercise), alpha of 0.05, statistical power of 0.80, and *r* of 0.90 [19]. To factor in possible dropouts, we initially recruited a sample of 18 participants. One participant dropped out due to private reasons; 17 participants (mean ± standard deviation (SD): age 23 ± 2 years, height 183 ± 5 cm, body mass 83 ± 11 kg) successfully completed all visits and were included in the analysis. Habitual caffeine intake of the participants was estimated using a validated food frequency questionnaire [20] and amounted to 67 ± 90 mg·day^−1^ (range: 0 to 357 mg·day^−1^). Of note here, only one participant had a high habitual caffeine intake of 357 mg·day^−1^; all remaining participants ingested <180 mg·day^−1^ with 12 ingesting <100 mg·day^−1^. Ethical approval was obtained from the Committee for Scientific Research and Ethics of the Faculty of Kinesiology at the University of Zagreb. Upon informing the participants about the study requirements, benefits, and risks, they provided written informed consent.

### 2.3. Exercise Tests

#### 2.3.1. Vertical Jump 

After the warm-up, the testing protocol started with the assessment of jump performance. The participants performed three squat jumps (SJs) and three countermovement jumps (CMJs) on the force platform (BP600600, AMTI, Inc., Watertown, MA, USA). The force platform was accompanied with a custom-developed software for data acquisition and analysis. Vertical jump height for both the SJ and the CMJ was automatically calculated by the software from the vertical velocity of the center of mass at take-off data using the following formula [21]:

$$vertical\;jump\;height\;=\;{TOV}^{2\;}/\;2g$$

where *TOV* is the vertical velocity of the center of mass at take-off, and *g* is the gravitational acceleration (9.81 m·sec^−2^).

The SJ was performed while starting from an initial semi-squat position (knees ~90° and trunk/hips in a flexed position), with participants holding the position for approximately 2 s before jumping vertically as quickly and as explosively as possible, in order to jump as high as possible in the shortest possible time using a concentric-only muscle action. Hands remained akimbo for the entire movement to eliminate any arm-swing influence. The participants were instructed to maintain fully extended lower limbs throughout the flight period. The CMJ was performed starting from the upright standing position. On the command of the tester, the participants performed a downward countermovement by a fast knee flexion. Immediately after, the vertical jump began by an explosive extension of the legs. The CMJ is characterized by an eccentric–concentric muscle action often referred to as the stretch-shortening cycle muscle action. The participants were instructed that their lowest position should be a semi-squat position (knees ~90° and trunk/hips in a flexed position), and that the jump should be performed as quickly and explosively as possible in order to jump as high as possible in the shortest possible time. One warm-up attempt for both the SJ and CMJ was allowed, during which the correct execution of the jumps was confirmed. Three official attempts followed, with 1 min of rest between the attempts; the highest jumps were used for the analysis. 

#### 2.3.2. Isokinetic Strength and Power 

The isokinetic dynamometer (System 4 Pro, Biodex Medical Systems, Inc., Shirley, NY, USA) was used for the isokinetic strength and power assessment of the knee extensor and knee flexor muscles. The assessment was performed unilaterally, involving only the dominant leg. The participants were placed in a seated position and stabilization straps were applied to the trunk, waist, thigh, and shin. The lateral femoral epicondyle of the dominant leg was aligned with the dynamometer’s axis of rotation. The isokinetic dynamometer was calibrated before each testing session, and the range of motion of the knee joint was set at 80°. Testing was performed at angular velocities of 60°·s^−1^ and 180°·s^−1^, in that order. At each angular velocity, participants first performed three familiarization repetitions to get accustomed to the speed of the lever arm. Then, following a 30-s rest interval, they performed five maximal knee extensions and flexions. For this exercise, the participants were instructed to extend and flex the knee (to “kick” and “pull”) five times as hard and as fast as they could. Peak torque in N·m^−1^ obtained during knee extension and knee flexion movement patterns was used as the measure of the knee extensor and knee flexor muscle strength, respectively. Average power over five repetitions at both angular velocities (i.e., 60°·s^−1^ and 180°·s^−1^) was also used for the analysis. 

#### 2.3.3. Bench Press 

The PowerLift mobile phone application was used to measure barbell velocity in the bench press exercise. The PowerLift application has previously been reported as valid, reliable, and accurate for measuring barbell velocity during this exercise [22]. The application allowed video recording of the lift in slow motion. After the recording was complete, the application allowed frame-by-frame inspection of the recorded video material and manual selection of the beginning and the end of the concentric part of the movement. The beginning of the movement was considered as the moment when the barbell left the chest of the participant. The end of the movement was considered as the moment when the participants fully extended the elbows. This distance (d) between the beginning and end of the movement was measured with a measuring tape and entered into the application. The application calculated the time (in ms) between two frames (i.e., the beginning and the end of the movement). The outcome of this test was the mean barbell velocity produced during the press. During each testing session, the participants exercised with loads corresponding to 50%, 75%, and 90% of their one-repetition maximum (1RM; established during the familiarization session), while completing two, one, and one repetition, respectively. During each repetition, the participants were instructed to perform the concentric part of the movement as fast as possible. Three minutes of rest were allowed between repetitions and/or loads.

#### 2.3.4. Rowing Ergometer Test

A test on a rowing ergometer (Model D, Concept II, Inc., Morrisville, VT, USA) was used to assess whole-body power. For this test, the resistance control dial of the ergometer was set at 10 (highest adjustable resistance). First, the participants were given 5 min during which they rowed comfortably at their own pace. No attempts were made to make any corrections in their rowing technique. Then, following a 2-min rest, the participants performed six “introductory” strokes, which were followed by six “all-out” strokes. For the six “all-out” strokes, the participants were instructed to row as hard and as fast as they could. The outcome of the test was peak power output, defined as the highest power output produced during the six “all-out” strokes (expressed in Watts), as shown on the performance monitor of the Concept II ergometer. This test has high test–retest reliability, and was previously validated by a group of physically active individuals by Metikos et al. [23], where it is explained in greater detail. 

### 2.4. Side Effects

Immediately following the completion of the exercise testing session and the morning after the testing, participants completed an eight-item survey regarding their subjective perceptions of side effects that may have occurred (“yes/no” response scale). This scale has been used in previous research that examined the effects of caffeine ingestion on exercise performance [14]. 

### 2.5. Assessment of Blinding 

We tested the effectiveness of the blinding pre- and post-exercise by asking participants to identify the supplement they had ingested. The question for identification went as follows: “Which supplement do you think you have ingested?” This question had three possible answers: (a) caffeine; (b) placebo; (c) do not know [24].

### 2.6. Statistical Analysis

A Shapiro–Wilk test was used to assess the normality of distribution. Upon confirming the normality of distribution, a series of one-way repeated measures ANOVAs was used to analyze the differences between conditions (i.e., placebo and caffeine) for all the performance outcomes. The statistical significance threshold was set at *p* < 0.05. Effect sizes (*d*) were calculated using a Cohen’s formula, in which the mean difference between the two measurements is divided by the pooled SD. Trivial, small, moderate, and large effect sizes were considered as <0.20, 0.20–0.49, 0.50–0.79, and ≥0.80, respectively [25]. Percent changes were also calculated. The effectiveness of the blinding was examined using Bang’s blinding index (BBI) where -1.0 indicates opposite guessing and 1 complete lack of blinding. A McNemar test was used to explore the differences in the incidence of side effects between the placebo and caffeine conditions. All analyses were performed using Statistica software (StatSoft; Tulsa, OK, USA).

## 3. Results

### 3.1. Exercise Tests

#### 3.1.1. Vertical Jump

Compared to placebo, caffeine ingestion improved performance both in the SJ (*p* = 0.039; *d* = 0.18; +2.9%) and in the CMJ (*p* = 0.011; *d* = 0.18; +3.3%). 

#### 3.1.2. Lower-Body Isokinetic Strength and Power

Caffeine ingestion had a significant effect on peak torque at the angular velocity of 60°·s^−1^, both in the knee extensor (*p* = 0.002; *d* = 0.37; +6.9%) and in the knee flexor muscles (*p* = 0.034; *d* = 0.24; +4.6%). At the angular velocity of 180°·s^−1^, caffeine ingestion elicited a significant effect on peak torque in the knee extensor (*p* = 0.031; *d* = 0.21; +3.5%), but not in the knee flexor muscles (*p* = 0.168; *d* = 0.17; +3.0). For average power, at the angular velocity of 60°·s^−1^, caffeine had a significant effect in increasing power both in the knee extensor (*p* = 0.001; *d* = 0.31; +6.3%) and the knee flexor muscles (*p* = 0.015; *d* = 0.32; +6.7%). At the angular velocity of 180°·s^−1^, a significant effect of caffeine on power produced by the knee extensor muscles was evident (*p* = 0.025; *d* = 0.25; +4.5%); however, the same was not the case for the knee flexor muscles (*p* = 0.115; *d* = 0.17; +3.5) (Table 2). 

#### 3.1.3. Bench Press

For barbell velocity in the bench press exercise, a significant effect of caffeine was observed at 50% of 1RM (*p* = 0.021; *d* = 0.33; +3.5%), at 75% of 1RM (*p* < 0.001; *d* = 0.42; +5.4%), as well as at 90% of 1RM (*p* < 0.001; *d* = 0.59; +12.0%).

#### 3.1.4. Rowing Ergometer Test

No significant effect of caffeine was observed for peak power output on the rowing ergometer test (*p* = 0.647; *d* = 0.08; +1.4).

### 3.2. Side Effects

The incidence of side effects is presented in Table 3. Based on the results of the McNemar test, none of the comparisons between the caffeine and placebo conditions were significant (*p* > 0.05 for all comparisons).

### 3.3. Assessment of Blinding

The results from the assessment of blinding pre- and post-exercise are presented in Table 4. When assessed pre-exercise, the BBI for the placebo and caffeine treatments amounted to 0.29 (95% confidence interval (CI): −0.06, 0.65), and 0.24 (95% CI: −0.07, 0.54), respectively. When assessed post-exercise, the BBI for the placebo and caffeine conditions amounted to 0.70 (95% CI: 0.49, 0.93) and 0.35 (95% CI: 0.00, 0.72), respectively. Those that correctly identified caffeine generally reported a “better overall feeling” and “more energy”, as well as increased perspiration. 

## 4. Discussion

The present study aimed to explore the effects of caffeinated gel ingestion on exercise performance of resistance-trained men in tests characterized by a very short duration and maximal exertion. The results indicate that caffeine ingestion in the form of a caffeinated gel had performance-enhancing effects on: (1) vertical jump performance in the SJ and CMJ tests; (2) lower-body isokinetic strength and power; and (3) power of the upper-body musculature. Whole-body power, as assessed on a rowing ergometer test, did not improve following caffeine ingestion. The blinding of the participants was generally effective, and the side effects were minimal. 

For the vertical jump performance, our results confirm the recent meta-analytical results by Grgic et al. [11] that caffeine ingestion before exercise may acutely enhance jump height. Indeed, even the effect size in the SJ and CMJ tests that we observed (*d* of 0.18 for both tests) were very similar to the pooled effect size of 0.17 reported in the meta-analysis. Previous studies that reported ergogenic effects of caffeine on jump performance generally used larger doses of caffeine (e.g., 6 mg·kg^−1^), as well as a protocol that included a waiting time of 60 min from ingestion to the initiation of the exercise testing [11]. Our results highlight that ingesting even a smaller dose of caffeine (300 mg; ~3.6 mg·kg^−1^) in the form of a caffeinated gel administered 10 minutes before exercise, may also be ergogenic. These findings mirror those of Bloms et al. [26] who also used both jump techniques and reported that ingesting 5 mg·kg^−1^ of caffeine improved performance both in the SJ and CMJ tests. 

A recent meta-analysis [12] reported that caffeine ingestion acutely increases strength, as assessed by an isokinetic dynamometer. Our results provide further support for these findings, given that we observed increases in peak torque following the ingestion of caffeine with *d* across angular velocities and muscle groups (i.e., knee extensors and knee flexors) ranging from 0.21 to 0.37, and corresponding percent changes ranging from +3.5% to +6.9%. While the ergogenic effects of caffeine were noted at both angular velocities for the knee extensor muscles, a significant effect of caffeine on the knee flexor muscles was observed only at the velocity of 60°·s^−1^. This divergent effect between muscle groups might be due to the lower level of muscle activation during maximal contractions at baseline in the knee extensor muscles [27]. This naturally occurring lower level of activation may provide a greater “room for improvement” in contraction force following the ingestion of caffeine in this muscle group. Smaller muscle groups may have a higher muscle activation level at baseline and, therefore, are less affected by caffeine ingestion [27]. Caffeine ingestion also improved average power, with a magnitude of improvement similar to that observed for muscle strength. 

The ergogenic effect of caffeine on barbell velocity in the bench press exercise was evident across all three employed loads with the effects ranging from small (*d* = 0.33; +3.5%) to moderate (*d* = 0.59; +12.0%). These results provide further support to findings of the previous studies that explored the effects of caffeine on barbell velocity. For example, Mora-Rodriguez et al. [15] reported that caffeine ingestion in a dosage of 3 mg·kg^−1^, ingested 60 min before exercise, enhanced barbell velocity in the bench press when using external loads amounting to 75% 1RM. 

Pallarés et al. [17] suggested that the effects of caffeine on power might be external load- and caffeine dose-dependent. In that study, caffeine ingested in low and moderate doses (3 and 6 mg·kg^−1^) enhanced barbell velocity in the bench press at loads corresponding to 25% and 50% of 1RM. However, when using loads of 75% of 1RM, only the doses of 6 and 9 mg·kg^−1^ were effective. At the highest load of 90% of 1RM, only 9 mg·kg^−1^ was effective. The findings presented herein are not in full agreement with the work by Pallarés et al. [17] given that, in the present study, an absolute dose of 300 mg (~3.6 mg·kg^−1^) was ergogenic for barbell velocity across all three loading schemes (including 90% of 1RM).

In contrast to the work by Pallarés et al. [17], the magnitude of effect in the present study increased with an increase in the load that the participants lifted (Table 2). The most pronounced effect across loading schemes, amounting to a +12.0% increase in barbell velocity, was evident for the load corresponding to 90% of 1RM. Based on these results, it seems that the effects of caffeine are more noticeable, at least for this exercise, when requirements for the contraction force are the highest. Given the direct importance of high barbell velocity in the development of power [28], our results suggest that individuals might consider supplementing with caffeine before exercise to achieve acute increases in barbell velocity and, subsequently, stronger stimuli for the development of muscle power.

We did not observe any significant differences between placebo and caffeine conditions in the whole-body power, as assessed by the peak power output produced during the “all-out” rowing ergometer test. Based on these results, it does not seem that caffeine ingestion is ergogenic for whole-body peak power output; however, this could be due to large inter-individual variation in response to caffeine ingestion [29], and therefore needs to be explored in future studies with larger sample sizes.

### 4.1. Mechanisms of Caffeine 

Caffeine produces its ergogenic effects by binding to adenosine receptors [30]. After binding to these receptors, caffeine blunts the fatiguing effects of adenosine and subsequently reduces perceived exertion. Indeed, there is substantial evidence that caffeine’s effect of reducing perceived exertion is one of the primary mechanisms for its ergogenic effect on aerobic endurance [31]. However, the ergogenic effect of caffeine on high-intensity, short-duration tests (such as those performed in the current study) may be related to the release of calcium from the sarcoplasmic reticulum, and the subsequent inhibition of its reuptake [30]. These actions may be associated with neuromuscular function changes, as well as increased contractile force in skeletal muscles [32]. For the readers interested, these mechanisms of caffeine are discussed in greater detail elsewhere [30].

### 4.2. Limitations

The limitations of this study include the following: (1) the sample consisted of trained young men, which limited the generalizability of these results to those who are untrained, of older age, or to women; (2) we did not measure plasma levels of caffeine and, therefore, the amount of caffeine absorbed is not entirely clear; (3) an absolute dose of caffeine was used, whereas a relative dose might have been more appropriate (of note here, an absolute dose was given due to the fixed amount of caffeine per 75-mg gel sachet). 

One additional limitation [33] might be that 12 out of 17 participants correctly identified the placebo condition post-exercise; as determined by the 95% CI of the BBI, this identification was not solely due to chance. It is likely that correct identification of the placebo condition in the post-exercise assessment was due to the lack of perceived improvements in performance (only one participant answered “yes” to the perception of improved performance item following the ingestion of placebo). This may especially be evident given the small number of individuals that correctly identified placebos in the pre-exercise evaluation. From that aspect, it is possible that pre-exercise responses are of greater importance than the answers obtained post-exercise. Additionally, based on the findings by Tallis et al. [34], an argument can be made that the correct identification of the placebo did not confound the results. In that study, the participants experienced similar improvements in isokinetic peak torque both when they were told that they were given caffeine and received a dose of caffeine, and when they were told that they ingested the placebo even though the capsule contained caffeine. While the placebo was identified beyond random chance in the post-exercise assessment, correct identification of caffeine in the post-exercise assessment can be attributed solely to chance, as there was a 95% CI overlap with the null value. These results further support an actual ergogenic effect of caffeine.

### 4.3. Practical Applications

Ingesting a caffeine dose of 300 mg in the form of caffeine gel 10 min before exercise may elicit an acute ergogenic effect on vertical jump height, muscle strength, and power in an isokinetic strength assessment, as well as barbell velocity in the bench press exercise. Due to these ergogenic effects, trained individuals may consider supplementing with caffeinated gels before exercise for acute increases in performance.

## 5. Conclusions

The ingestion of caffeinated gels with an absolute dose of caffeine of 300 mg may improve aspects of short-term, maximal-exertion exercise performance in resistance-trained men. These improvements are evident in vertical jump performance, strength, and power. These results highlight that individuals seeking acute performance enhancement in jumping, strength, and power may consider ingesting caffeinated gels before exercise.

## Figures and Tables

**Table 1:** Test–retest reliability of the exercise protocol; determined on a pilot sample of five participants.

Exercise Test	Outcome	Average CV
Squat jump (SJ)	Jump height (cm)	1.3%
Countermovement jump (CMJ)	Jump height (cm)	1.3%
Isokinetic knee extension at 60° s^−1^	Peak torque (Nm)	2.5%
	Average power (W)	1.7%
Isokinetic knee flexion at 60° s^−1^	Peak torque (Nm)	5.3%
	Average power (W)	4.4%
Isokinetic knee extension at 180° s^−1^	Peak torque (Nm)	2.1%
	Average power (W)	2.7%
Isokinetic knee flexion at 180° s^−1^	Peak torque (Nm)	5.9%
	Average power (W)	5.0%
Bench press at 50% 1RM	Barbell velocity (m·s^−1^)	1.7%
Bench press at 75% 1RM	Barbell velocity (m·s^−1^)	3.6%
Bench press at 90% 1RM	Barbell velocity (m·s^−1^)	5.1%
Rowing ergometer test	Peak power (W)	2.5%

1RM: one-repetition maximum; CV: coefficient of variation.

**Table 2:** Differences in placebo vs caffeine conditions for the performance outcomes.

Exercise Test	Outcome	Caffeine Condition (mean ± SD)	Placebo Condition (mean ± SD)	*d* (95% CI)	Relative Effects (%)	*p*
Squat jump	Jump height (cm)	31.9 ± 4.9	31.0 ± 5.5	0.18 (0.03, 0.32)	+2.9	0.039 *
Countermovement jump	Jump height (cm)	36.4 ± 6.5	35.2 ± 6.5	0.18 (0.05, 0.32)	+3.3	0.011 *
Isokinetic knee extension at 60° s^−1^	Peak torque (Nm)	256.9 ± 44.5	240.3 ± 45.6	0.37 (0.15, 0.61)	+6.9	0.002 *
	Average power (W)	193.8 ± 36.9	182.3 ± 36.8	0.31 (0.13, 0.50)	+6.3	0.001 *
Isokinetic knee flexion at 60° s^−1^	Peak torque (Nm)	147.1 ± 24.6	140.7 ± 28.7	0.24 (0.02, 0.46)	+4.6	0.034 *
	Average power (W)	118.7 ± 21.0	111.3 ± 25.9	0.32 (0.01, 0.59)	+6.7	0.015 *
Isokinetic knee extension at 180° s^−1^	Peak torque (Nm)	180.2 ± 27.8	174.0 ± 31.9	0.21 (0.02, 0.40)	+3.5	0.031 *
	Average power (W)	353.4 ± 57.0	338.1 ± 66.6	0.25 (0.04, 0.46)	+4.5	0.025 *
Isokinetic knee flexion at 180° s^−1^	Peak torque (Nm)	110.0 ± 18.1	106.8 ± 20.3	0.17 (−0.07, 0.40)	+3.0	0.168
	Average power (W)	212.9 ± 38.0	205.8 ± 46.7	0.17 (−0.04, 0.38)	+3.5	0.115
Bench press at 50% 1RM	Barbell velocity (m·s^−1^)	0.83 ± 0.08	0.80 ± 0.09	0.33 (0.06, 0.61)	+3.5	0.021 *
Bench press at 75% 1RM	Barbell velocity (m·s^−1^)	0.57 ± 0.06	0.54 ± 0.07	0.42 (0.21, 0.64)	+5.4	< 0.001 *
Bench press at 90% 1RM	Barbell velocity (m·s^−1^)	0.39 ± 0.07	0.35 ± 0.07	0.59 (0.27, 0.89)	+12.0	< 0.001 *
Rowing ergometer test	Peak power (W)	725.4 ± 133.5	715.4 ± 106.4	0.08 (−0.27, 0.43)	+1.4	0.647

SD: standard deviation; *d*: effect size; CI: confidence interval; * denotes statistically significant differences.

**Table 3:** Incidence of side effects reported immediately after and the morning after ingestion of a caffeinated gel or a placebo.

	Placebo	Caffeine	Placebo	Caffeine
	Immediately After Testing Session	Immediately After Testing Session	Morning After Testing Session	Morning After Testing Session
Muscle soreness	0	0	0	0
Increased urine production	0	6	0	6
Tachycardia and heart palpitations	6	12	0	0
Increased anxiety	0	18	0	0
Headache	0	0	0	0
Abdominal/gut discomfort	0	6	0	0
Insomnia	n/a	n/a	0	6
Increased vigor/activeness	12	41	0	0
Perception of improved performance	6	35	n/a	n/a

Data are frequencies for 17 participants, expressed as the percentage of positive cases; none of the comparisons were significant based on the McNemar test.

**Table 4:** Results of the assessment of blinding pre- and post-exercise.

Pre-Exercise				
Condition	Responded as Placebo	Responded as Caffeine	Responded as Do not Know	Bang’s Blinding Index (Mean and 95% CI)
Placebo	6	2	9	0.29 (−0.06, 0.65)
Caffeine	3	8	6	0.24 (−0.07, 0.54)
**Post-Exercise**				
Placebo	12	0	5	0.70 (0.49, 0.93)
Caffeine	3	9	5	0.35 (−0.00, 0.72)

CI: confidence interval.
